# William Henry Stevens, M.R.C.S., L.R.C.P. Lond., L.S.A.

**Published:** 1913-09

**Authors:** 


### Obituary.

### WILLIAM HENRY STEVENS, M.R.C.S., L.R.C.P. Lond., L.S.A.

He was born in 1862, and died in 1913. Educated at the Bristol Grammar School, he afterwards became a student at the Medical School of University College, Bristol. After becoming qualified in 1886, he entered into partnership with Dr. Roden, of Droitwich, and was soon appointed as Medical Officer of Health to the Borough of Droitwich. He thus became familiar with the methods of the hydrologists, but was never greatly impressed by the therapeutic potency of the high gravity saline baths of that town. After three years he returned to Bristol, and became medical officer to several societies, including the Foresters and the Clifton Friendly Society, and established a surgery at Dowry Parade. His diagnostic powers were quite good, and he was much appreciated by a large clientèle of patients. Recently he had become much interested in the occult sciences, and had great belief in suggestive treatment in nervous affections, insomnia and melancholia.

As amusements he once took up photography with considerable success, and for many years was a keen member of the Rodway Golf Club.

His end came in a somewhat tragic manner, for whilst bathing in the sea at Weston-super-Mare, on August 2nd, his daughter saw him fall down in shallow water. He picked himself up, gave a weird laugh, and fell down again. He was then dragged out in an unconscious condition with right hemiplegia, and he never spoke again or showed any sign of consciousness. His kind and sympathetic nature endeared him to a large circle of patients and friends, who will long remember his devotion to their welfare, and will regret that his useful life should be thus curtailed at the comparatively early age of fifty-one.

